# Comprehensive Transcriptomic and Metabolomic Analysis of the *Litopenaeus vannamei* Hepatopancreas After WSSV Challenge

**DOI:** 10.3389/fimmu.2022.826794

**Published:** 2022-02-10

**Authors:** Dianjiang Yu, Yufeng Zhai, Peimin He, Rui Jia

**Affiliations:** College of Marine Ecology and Environment, Shanghai Ocean University, Shanghai, China

**Keywords:** WSSV, *Litopenaeus vannamei*, transcriptome, metabolomics, immunity

## Abstract

*Litopenaeus vannamei* is the major farmed shrimp species worldwide. White spot disease due to white spot syndrome virus (WSSV) is severely affecting shrimp worldwide, causing extensive economic losses in *L. vannamei* culture. This is the first study that applied combined transcriptomic and metabolomic analysis to study the effects on the *L. vannamei* hepatopancreas after WSSV challenge. Our transcriptomic data revealed differentially expressed genes (DEGs) associated with immunity, apoptosis, the cytoskeleton and the antioxidant system in the hepatopancreas of *L. vannamei*. Metabolomic results showed that WSSV disrupts metabolic processes including amino acid metabolism, lipid metabolism and nucleotide metabolism. After challenged by WSSV, immune-related DEGs and differential metabolites (DMs) were detected in the hepatopancreas of *L. vannamei*, indicating that WSSV may damage the immune system and cause metabolic disorder in the shrimp. In summary, these results provide new insights into the molecular mechanisms underlying *L. vannamei*’s response to WSSV.

## 1 Introduction

*Litopenaeus vannamei*, a prawn species commonly farmed worldwide, is valued for its high nutritional value and relatively low price (1), and it provides large economic benefits to farmers (2). However, multiple pathogens, including white spot syndrome virus (WSSV), *Vibrio parahaemolyticus* taura, syndrome virus and *Vibrio harveyi* (1), have long affected the production of *L. vannamei*. Among them, WSSV is the most harmful virus in shrimp farming worldwide; it has a wide range of hosts, such as crabs, shrimp and crayfishes (2). However, no effective solution has been found to prevent and control WSSV.

Like other crustaceans, *L. vannamei* mainly relies on the innate immune system in dealing with pathogen invasion. As the most important organ of the immune system, the hepatopancreas plays a vital role in the immune response of shrimp (3). With the help of expressed sequence tag (EST) analysis and gene discovery technology, Gross et al. (4) found that hepatopancreas plays a key role in the innate immunity of *L. vannamei* and *L. setiferus* (4). In recent years, omics technologies such as genomics, transcriptomics, proteomics and metabolomics have become important means of biomarker screening, disease diagnosis and pathogenesis research (5–7). Among these, metabolomics combined with transcriptomics can help understand the metabolic changes in organisms (8, 9). Fan et al. (10) showed that several metabolites (Such as glucose, alanine, fumarate, phosphorylcholine, betaine and uracil) associated with important metabolic pathways are up-regulated in the gills and hepatopancreas of *Cherax quadricarinatus* infected with WSSV (10). Zhong et al. (3) showed that after WSSV challenge, the DEGs identified in *Marsupenaeus japonicus* hepatopancreatic transcriptome data mainly focused on JAK-STAT signaling pathway, Ras signaling pathway, integrin-mediated signal transduction, phagocytosis and apoptosis (3). Jin et al. (11) combined transcriptomic and metabolomic data, and found that different temperature and light conditions significantly affect the key genes and metabolites associated with the differentiation and development of *Macrobrachium nipponense* (11).

In this study, non-targeted metabolomics combined with transcriptomics was used to identify the differentially expressed genes (DEGs) and differential metabolites (DMs) in the hepatopancreas of *L. vannamei* 2 h, 36 h and 48 h after infection with WSSV to explore the possible mechanism underlying their functions and relationships with immunity, and to provide a reference for the immune response mechanism of *L. vannamei* at different times after WSSV infection.

## 2 Materials and Methods

### 2.1 WSSV and Shrimp Culture

The virus strain was the Chinese mainland strain (WSSV-CN, AF-332093). WSSV virus liquid (2 × 10^8^ copies/μl) was donated by the Third Institute of Oceanography of the State Oceanic Administration (12). *Macrobrachium nipponense* was purchased from Shanghai Guoyuan. After being temporarily reared in a laboratory environment for 5–7 days, healthy *M. nipponense* were injected intramuscularly with 50μl WSSV virus solution. Two days later, dead *M. nipponense* were randomly collected, and polymerase chain reaction (PCR) was used to verify WSSV-infection (13). The accumulated amount of virus in the *M. nipponense* we fed was ranged from 10^3^ to 10^5^/ng DNA. Only tissues from infected *M. nipponense* were fed to *L. vannamei*.

### 2.2 Daily Management and Experimental Design

*L. vannamei* was purchased from a shrimp farm in Shanghai Guoyuan without specific pathogens, and the body length and weight of each shrimp are 4cm and 1 ± 0.05g, respectively. The shrimp were temporarily reared for 5 days. During temporary rearing, 1/3 of the water was changed every day, and the water was maintained at 28°C and a salinity of 5‰. The water quality was monitored every day, the ammonia nitrogen content was below 0.5mg/L, and the nitrite content was below 0.2mg/L. Shrimp were fed commercial bait (crude protein ≥ 48.00%, crude fat ≥ 7.0%, crude fiber ≤ 5.0%, crude ash ≤ 16.0%, the main raw materials of feed are fish meal and krill meal) two times per day. After that, 1800 shrimp were starved for 24 h and divided into two groups. Shrimp in the experimental group (P) were fed infected *M. nipponense* at 5% body weight (14); and those in the negative control group (N) were fed healthy *M. nipponense* in the same amount. Each group had three replicates, each comprising 300 shrimp. The breeding conditions during the experiment were the same as those during the temporary period. At 2h, 36h and 48h (P2, P36, P48) after the infected *M. nipponense* was fed to *L. vannamei*, we randomly selected 20 shrimp in each replicate of the experimental group to remove the hepatopancreas with sterile forceps, and stored at −80°C for further analysis. A total of 60 shrimp were randomly selected for three time points of each replicate. During the infection period, dead *L. vannamei* were randomly collected for PCR to verify WSSV-infection (**Figure S1**).

### 2.3 Transcriptomic Analysis

#### 2.3.1 RNA Extraction, Library Preparation and Illumina Hiseq xten Sequencing

Total RNA in shrimp hepatopancreas tissue was extracted with TRIzol (Invitrogen) according to the manufacturer’s protocol and genomic DNA was removed using DNase I (TaKara). Then, with a 2100 Bioanalyzer (Agilent) and ND-2000 spectrophotometer (NanoDrop Technologies), the quantity and quality of the extracted total RNA were tested to validate the RNA samples. Only high-quality RNA sample (OD260/280 = 1.8~2.2, OD260/230≥2.0, RIN≥6.5, 28S:18S≥1.0, >2μg) was used to construct sequencing library. RNA-seq transcriptome librariy was prepared following TruSeq™ RNA sample preparation Kit from Illumina (San Diego, CA) using 1μg of total RNA. According to the principle of A-T base pairing between magnetic beads with oligo (dT) and the polyA at the 3’ ends of mRNA, mRNA can be separated from total RNA for transcriptomic analysis. To randomly break large fragments of mRNA into 300 bp fragments, we added fragmentation buffer under suitable conditions. Then the mRNA was reverse transcribed into cDNA; after being connected to the adaptor, the mRNA was purified and amplified by PCR to obtain the final library. Finally, paired-end RNA-seq sequencing library was sequenced with the Illumina HiSeq xten (2 × 150bp read length).

#### 2.3.2 Read Mapping

To obtain high-quality clean data to ensure the accuracy of subsequent analysis results, we used SeqPrep (https://github.com/jstjohn/SeqPrep) and Sickle (https://github.com/najoshi/sickle) to perform quality control on the original test data. Then use TopHat (http://ccb.jhu.edu/software/tophat/index.shtml, version v2.1.1) software to align the clean reads with the reference genome. The genome of *L. vannamei* (Reference genome version: ASM378908v1; https://www.ncbi.nlm.nih.gov/genome/10710?genome_assembly_id=422001) was selected as the reference genome, and the comparison rate ranged from 81.7% to 90.0%. The mapping criteria of bowtie was as follows: the sequencing reads should uniquely match the genome, allowing up to 2 mismatches, without insertion or deletion. Then the region of gene were expanded according to the depth of the site and obtain the operon. In addition, the entire genome is divided into multiple 15kbp windows sharing 5kbp. The newly transcribed region is defined as more than 2 consecutive windows without overlapping gene regions, where at least 2 reads in each window are mapped in the same direction.

#### 2.3.3 Identification of Differentially Expressed Genes

To identify the differentially expressed genes between the samples, we calculated the expression level of each transcript according to the fragments per kilobase of exons per million mapped reads (FRKM) method. RSEM (http://deweylab.biostat.wisc.edu/rsem/, version 1.3.3) is used to quantify gene abundance. R statistical package software EdgeR (http://www.bioconductor.org/packages/stats/bioc/edgeR/, version 3.14.0) was utilized for differential expression analysis. In addition, GO annotation analysis and KEGG function enrichment analysis (P-adjust ≤ 0.05) are performed by Blast2go (https://www.blast2go.com/, version 2.5) and KOBAS (http://kobas.cbi.pku.edu.cn/download.php, version 2.1.1), respectively.

#### 2.3.4 Quantitative Real-Time PCR Verification

To further verify the reliability and accuracy of the transcriptomic data, we used quantitative real-time PCR (qRT-PCR) for verification. Alkaline phosphatase-like (ALP-like), trypsin-1-like, C-type lectin domain family 4 member G-like, transcript variant X1 (Clec4g-like, transcript variant X1), crustacyanin-C1 subunit-like, phosphoenolpyruvate carboxykinase, cytosolic [GTP]-like, transcript variant X1 (PCK, cytosolic [GTP]-like, transcript variant X1), protein argonaute-3-like (AGO-3-like) and chymotrypsin BI-like(CHT BI-like) were selected for qRT-PCR analysis. **Table 1** shows the primers designed for the seven genes. The cDNA was synthesized with HiScript Q RT SuperMix for qPCR with gDNA wiper (Vazyme, China). qRT-PCR was performed on an ABI7300 fluorescence quantitative PCR instrument (Bio-Rad, USA) with ChamQ SYBR Color qPCR (Vazyme, China) according to the manufacturer’s recommendations. All reactions were performed in triplicate. The expression level of the target gene in each sample was quantified by the relative fold change normalized to β-actin with the 2^−ΔΔCT^ method.

**Table 1:** The primer sequences used for qRT-PCR.

Gene name	Sequence (5′-3′)	Product size (bp)
ALP-like	F:AAGAGCCTGAACCTGGAC	270
	R:GGGATAGCCACTGATAGACA	
trypsin-1-like	F:GACAGCCACTACGACGAC	262
	R:ACAGGAACCAAGGGAACA	
Clec4g-like, transcript variant X1	F:AAGGCGTCAGCGACAATG	262
	R:ACCGACCGTTTCTCCAAA	
crustacyanin-C1 subunit-like	F:TCTATGAGCAGCAAAGGC	281
	R:CTCGTAGTCGGTGTCCAG	
PCK, cytosolic [GTP]-like, transcript variant X1	F:CGCAGCAGAGCACAAAGG	202
	R:ATTCTCCCCGAAACCAGG	
AGO-3-like	F:CTTAGTGATGGTCGTGCG	296
	R:GGTTTAATCTCCTTGCCG	
CHT BI-like	F:TTCACCCACGAGGATTGG	246
	R:ATACACGGCGTCGCAGTC	
β-actin	F:TTTGCGACTCTGGTGATGGT	157
	R:GCGGTGGTGGTGAAAGAATAG	

### 2.4 Metabolomic Analysis

#### 2.4.1 Sample Extraction and Detection Methods

A total of 50 mg of each sample was accurately weighed and transferred to a 2 mL centrifuge tube containing one small steel ball. Then 400 μL of the extraction (solvent methanol/water: 4/1, v/v) and 20 μL of the internal standard (2-chloro-L-phenylalanine) at a concentration of 0.02 mg/mL were added to the centrifuge tube. After 6 minutes of grinding in a frozen tissue grinder (-10°C, 50 Hz) and 30 minutes of low-temperature ultrasonic extraction (5°C, 40 KHz), the sample was allowed to stand at -20°C for 30 minutes. Finally, the sample was centrifuged for 15 minutes, and the supernatant was transferred to a sample vial for LC-MS detection. Quality control samples were used to ensure the stability of the sample detection process. These samples were made up of equal volumes of extracts from all samples.

#### 2.4.2 Data Processing and Metabolite Identification

The sample was separated by liquid chromatography, and the single components entered the ion source of the high-vacuum mass spectrometer for ionization. The mass-to-charge ratio (m/z) was used to obtain the mass spectrum. Finally, the mass spectrum data of the sample were analyzed to obtain the qualitative and quantitative results. The original data included quality control samples and test samples. To better analyze the data, we preprocessed the original data. First, the 80% rule was used to remove missing values (low-quality peaks), and then the missing values were replaced with minimum values. To eliminate systematic errors, such as sample and instrument deviations, we used sum normalization to unify the data. QC samples with standard deviations greater than 30% were eliminated, and finally log_10_ logarithmic processing was used to make the data follow a normal distribution and obtain the final data matrix for subsequent analysis. The raw data was imported into the metabolomics processing software ProgenesisQI (Waters Corporation, Milford, USA) for processing. Later, the software was used to search and identify the characteristic peaks, and the mass spectrometry information was matched with the metabolic database (http://www.hmdb.ca/ and https://metlin.scripps.edu/), and the metabolites were identified based on the matching score of the secondary mass spectrum.

#### 2.4.3 Statistical Analysis

Principal component analysis (PCA) and partial least squares discriminant analysis (PLS-DA) were performed on the final data, The PCA model diagram generated by seven-fold cross-validation and the PLS-DA model diagram obtained with 200× response permutation testing were used to evaluate the stability of the model, and t-tests were performed. According to VIP > 1 and p < 0.05, DMs with statistically significant differences between the groups were selected.

## 3 Results

### 3.1 Transcriptomic Analysis of the Hepatopancreas of *L. vannamei* After WSSV Challenge

#### 3.1.1 Transcriptomic Data Sequencing and Quality Control

Transcriptomic analysis was performed on the hepatopancreas of *L. vannamei*, and 591,244,356 raw reads and 89,277,897,756 raw bases were obtained. After data quality control, a total of 585,442,166 clean reads and 87,202,960,063 clean bases were obtained. The proportion of each sample with quality score ≥ Q20 and ≥ Q30 exceeded 98% and 96%, thus indicating the high quality of reads (**Table 2**). The GC content of each sample exceeded 48.56%.

**Table 2:** Sequencing data statistics. Raw reads and raw bases represent the total number of entries and total data volume of the original sequencing data, respectively.

Groups	Sample	Raw reads	Raw bases	Clean reads	Clean bases	Q20(%)	Q30(%)	GC content(%)
N	N-1	47276166	7138701066	46780920	6986051239	98.9	96.46	48.56
	N-2	51570250	7787107750	51120258	7623801433	98.98	96.67	50.16
	N-3	45558740	6879369740	45014318	6705757844	98.85	96.3	49.41
P2	P2-1	44104964	6659849564	43702724	6522557569	98.97	96.63	49.1
	P2-2	51890530	7835470030	51446076	7685343418	98.97	96.65	49.75
	P2-3	45419602	6858359902	44945744	6705021273	98.86	96.32	49.38
P36	P36-1	46438140	7012159140	45999222	6868260256	99.02	96.74	49.52
	P36-2	54986172	8302911972	54438318	8111072017	98.94	96.55	49.06
	P36-3	54883354	8287386454	54338752	8118140156	98.97	96.66	49.08
P48	P48-1	46351256	6999039656	45823224	6784292256	98.96	96.62	48.81
	P48-2	54964170	8299589670	54493036	8085992898	99.06	96.91	50.4
	P48-3	47801012	7217952812	47339574	7006669704	98.93	96.46	49.6

Clean reads and clean bases represent the total number of entries and total data volume of the sequencing data after quality control, respectively.

#### 3.1.2 Identification of Differentially Expressed Genes

**Figure 1:**
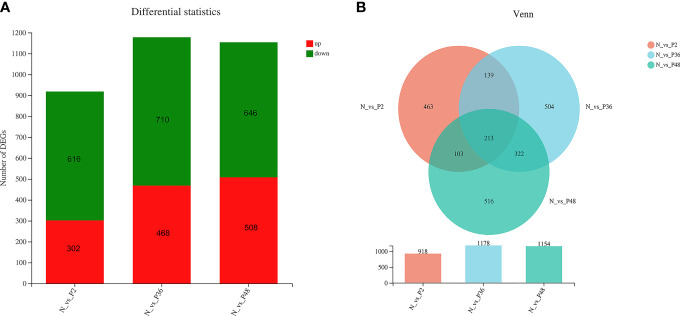
Differentially expressed gene (DEG) analysis. **(A)** DEGs in N vs P2, N vs P36, and N vs P48. **(B)** Venn diagram of comparison of DEGs.

The expression levels of unigenes were compared in shrimp between each time point after challenge (P2, P36 and P48) and the N group to identify the DEGs. A total of 918 (including 302 up-regulated genes and 618 down-regulated genes), 1178 (including 468 up-regulated genes and 710 down-regulated genes) and 1154 (including 508 up-regulated genes and 646 down-regulated genes) DEGs were obtained in N vs P2, N vs P36, and N vs P48, respectively (**Figure 1A**). We performed detailed analysis of the up-regulated and down-regulated DEGs of the three pairwise comparison groups (**Tables S1** **–** **S6**). In three pairwise comparisons of N vs P2, N vs P36, and N vs P48, we identified the common DEGs. A total of 213 common genes were identified in three pairwise expression analyses, as shown in the Venn diagram (**Figure 1B**). Among the 213 DEGs, there are 81 up-regulated genes and 132 down-regulated genes in N vs P2, 82 up-regulated genes and 131 down-regulated genes in N vs P36 and 81 up-regulated genes and 132 down-regulated genes in N vs P48.

#### 3.1.3 GO and KEGG Enrichment Analysis of Differentially Expressed Genes

**Figure 2:**
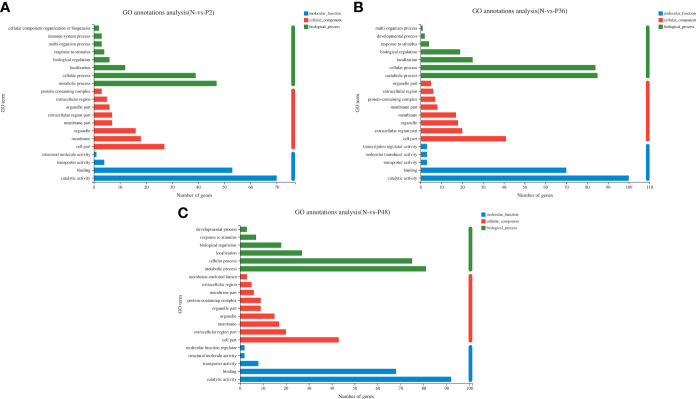
Gene ontology (GO) annotation analysis of DEGs in pairwise comparisons between the control (N) and different time points after challenge (P2, P36 and P48). **(A)** N vs P2. **(B)** N vs P36. **(C)** N vs P48.

To further explore the immune-related genes and pathways in the hepatopancreas of *L. vannamei* after WSSV challenge, we performed GO annotation analysis (**Figure 2**). DEGs were divided into three categories: molecular function, cellular component and biological process. In the molecular function category, most DEGs were associated with catalytic activity and binding. In the cellular component category, the dominant subcategories were categorized into cell part, extracellular region part, organelle, membrane and membrane part. Among the biological processes, the categories of metabolic process, cellular process, localization and biological regulation were most enriched; the DEGs in the three experimental groups were also concentrated in the above-mentioned functional categories. Notably, 36 h after WSSV challenge, the enrichment in the main subtypes of the three categories all reached a maximum.

**Figure 3:**
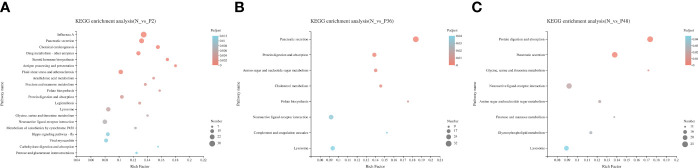
Kyoto Encyclopedia of Genes and Genomes (KEGG) analysis of DEGs in pairwise comparisons between the control (N) and different time points after challenge (P2, P36 and P48). **(A)** N vs P2. **(B)** N vs P36. **(C)** N vs P48.

In addition, KEGG pathway enrichment analysis was performed to identify sets of DEGs involved in the enrichment of specific pathways. A total of 434, 564 and 531 DEGs were annotated to 271, 286 and 296 signaling pathways in N vs P2, N vs P36, and N vs P48, respectively. Detailed information on the signal pathway enrichment can be found in **Tables S7** **–** **S9**. The most enriched pathways of DEGs in the P2 compared with the N group were influenza A, pancreatic secretion, fluid shear stress and atherosclerosis, neuroactive ligand-receptor interaction, lysosome, drug metabolism—other enzymes, viral myocarditis and chemical carcinogenesis (**Figure 3A**). In the N-P36 group, DEGs were mainly enriched in pancreatic secretion, neuroactive ligand-receptor interaction and lysosome (**Figure 3B**). In the N-P48 group, DEGs in protein digestion and absorption, pancreatic secretion, neuroactive ligand-receptor interaction and lysosome were significantly enriched (**Figure 3C**).

#### 3.1.4 Differentially Expressed Genes in the Hepatopancreas of *L. vannamei* After WSSV Challenge

On the basis of the NR database annotations, several DEGs were identified, which provided a better understanding of the hepatopancreas autoimmune response of *L. vannamei* after WSSV challenge. Among the 213 DEGs shared by the three pairwise comparison groups, many were identified to be associated with the immune system, apoptosis and cytoskeleton, such as C-type lectin domain family 6 member A-like, cathepsin L1-like, chitinase-3-like protein 1, G-protein coupled receptor Mth-like, mucin-5AC-like, caspase-1-A-like, ceramide synthase 2-like, transcript variant X2, E3 ubiquitin-protein ligase RNF216-like, programmed cell death protein 6-like, serine/threonine-protein kinase fray2-like, zonadhesin-like, actin-2, muscle-specific-like, transcript variant X2 and actin cytoskeleton-regulatory complex protein pan-1-like. The DEGs associated with metabolic processes included phospholipase D alpha 1-like, phosphatidate phosphatase LPIN3-like, phospholipid-transporting ATPase IF and trypsin-1-like were mainly involved in lipid metabolism and amino acid metabolism. In addition, some DEGs involved in the detoxification antioxidative system were observed, such as cytochrome P450 2L1-like, cytochrome P450 4C1-like, liver carboxylesterase 2-like, glutathione S-transferase 1-like, peroxisomal N(1)-acetyl-spermine and superoxide dismutase [Cu-Zn]-like (SOD) (**Table S10**). Similarly, many unique DEGs in the P2, P36 and P48 groups, compared with the N group, were involved in immune system, apoptosis, detoxification, the cytoskeleton and the antioxidant system (**Tables S11** **–** **13**).

### 3.2 qRT-PCR Validation of DEGs

**Figure 4:**
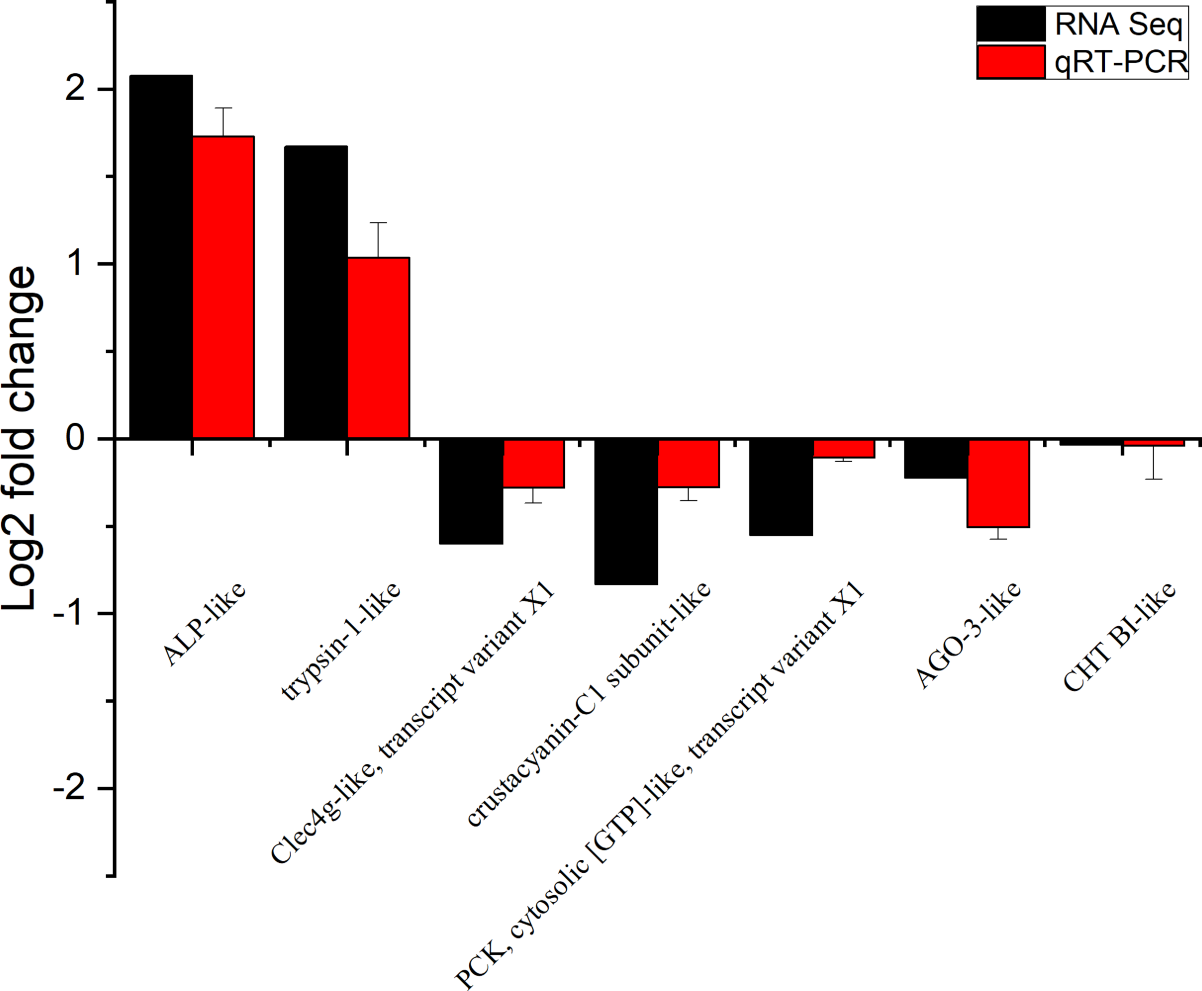
Expression of seven differentially expressed genes (DEGs) in the transcriptome, verified by qRT-PCR. Beta-actin was used as an internal reference gene to standardize the data, and fold changes are shown to verify the RNA-seq results. ALP-like (alkaline phosphatase-like); Clec4g-like, transcript variant X1 (C-type lectin domain family 4 member G-like, transcript variant X1); PCK, cytosolic [GTP]-like, transcript variant X1 (phosphoenolpyruvate carboxykinase, cytosolic [GTP]-like, transcript variant X1); AGO-3-like (protein argonaute-3-like); CHT BI-like (chymotrypsin BI-like). Vertical bars represented the mean ± S.D. (n = 3).

To verify the accuracy of RNA-seq detection, seven DEGs were randomly selected for qRT-PCR verification. The expression patterns of all seven genes were very similar to the RNA-seq results, thus further illustrating the reliability of the transcriptomic data (**Figure 4**).

### 3.3 Differential Metabolite Analysis of Non-Targeted Metabolomics

#### 3.3.1 QC Sample Principal Component Analysis

**Figure 5:**
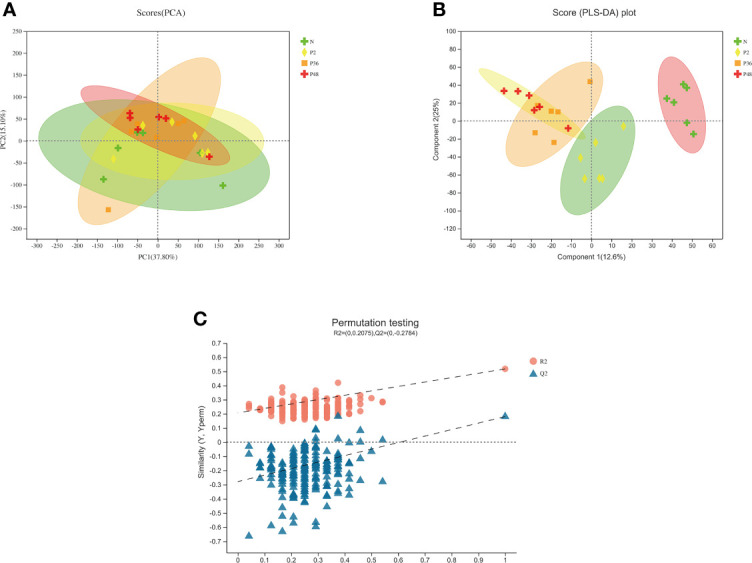
Quality analysis chart of metabolomics data of the *L. vannamei* hepatopancreas at different times after WSSV challenge. **(A)** Principal component analysis (PCA) score chart. The parameters of the first and second principal components R^2^X (cum) were 0.378 and 0.53, respectively. **(B)** Partial least-squares discriminant analysis (PLS-DA) score plot. **(C)** A total of 200 permutation tests on PLS-DA were performed to obtain model verification of PLS-DA. The parameters of the R2 and Q2 scores were 0.2075 and −0.2784, respectively.

In this study, non-targeted metabolomic methods were used to analyze the DMs of the hepatopancreas in *L. vannamei* among the N-P2, N-P36 and N-P48 groups after WSSV challenge, to determine the possible changes in DMs at different times after WSSV infection. The tight arrangement of the samples in PCA analysis indicated that the experiment was reproducible (**Figure 5A**). The PLS-DA model evaluation parameters R^2^X (cum) = 0.376, R^2^Y (cum) = 0.518, Q^2^ = 0.16 indicated that the model was stable and reliable (**Figure 5B**), and the slopes of the non-parallel straight lines of R^2^ and Q^2^ indicated that the PLS-DA model was not overfitted (**Figure 5C**).

#### 3.3.2 Identification of Differential Metabolites and Enrichment Analysis of KEGG Metabolic Pathway

**Figure 6:**
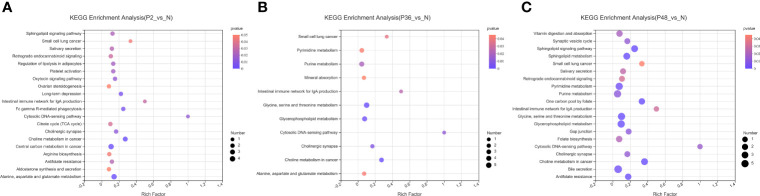
KEGG pathway enrichment analysis of the hepatopancreas of *Litopenaeus vannamei* at different times after WSSV challenge. **(A)** KEGG pathways of P2 vs N. **(B)** KEGG pathways of P36 vs N. **(C)** KEGG pathways of P48 vs N. The abscissa of the bubble chart is the enrichment rate, and the ordinate is the KEGG pathway. The bubble size represents the amount of metabolites enriched in the metabolic concentration in the pathway. The bubble color indicates the size of the P value indicating enrichment significance.

In this study, non-targeted metabolomics was used to analyze the differential metabolites of *L. vannamei* in the hepatopancreas at different times after WSSV infection. The results showed that compared with the N group, the number of different metabolites in the three experimental groups (P2, P36 and P48) were 353, 332, and 405, respectively (**Figure S2**). To explore the potential metabolic pathways affected by WSSV challenge in *L. vannamei* after 2 h, 36 h and 48 h, we further analyzed all DMs on the basis of KEGG annotations. The most enriched pathways in the P2 group compared with the N group were alanine, aspartate and glutamate metabolism; central carbon metabolism in cancer; and choline metabolism in cancer (**Figure 6A**). The most enriched pathways in the P36 group were glycine, serine and threonine metabolism; glycerophospholipid metabolism; and purine metabolism (**Figure 6B**). The most enriched pathways in the P48 group were bile secretion; glycerophospholipid metabolism; glycine, serine and threonine metabolism; purine metabolism; and pyrimidine metabolism (**Figure 6C**). With increasing time after WSSV challenge, the levels of glycerophospholipid metabolism; glycine, serine and threonine metabolism; and purine metabolism in the hepatopancreas of the experimental group of *L. vannamei* also increased significantly.

### 3.4 Conjoint Transcriptomic and Metabolomic Analysis

Finally, to determine the correlation between the transcriptomic and metabolomic data, we performed correlation network analysis of DMs and DEGs with Pearson correlation coefficients. In the P2 group, compared with the N group, Ne-methyl-L-lysine, 6-(alpha-D-glucosaminyl)-1D-myo-inositol, adenine, methylpyrazine, niacinamide, citrulline, arginyl-phenylalanine and lysinoalanine were positively correlated in the transcriptome and metabolome (**Tables S14-A**). In P36, glutaminyltryptophan, glycitein, nicotinic acid mononucleotide, 3-methyldioxyindole, N-methoxycarbonyl-N-nornuciferine, 2-(2-aminoethyl)indole, pyrocatechol sulfate, D-ornithine, N6-galacturonyl-L-lysine and S-(2-methylbutanoyl)-dihydrolipoamide were positively correlated (**Tables S14-B**). In P48, guanine, leucyl-phenylalanine, dihydromaleimide beta-D-glucoside, arabinonic acid, dopamine, romucosine D, pyrocatechol sulfate, glutaminyltryptophan, folic acid, daidzein, nicotinic acid mononucleotide, phenylalanyl-tryptophan, asparaginyl-hydroxyproline and aspartyl-gamma-glutamate were positively correlated (**Tables S14-C**).

## 4 Discussion

With the increase in the production of *L. vannamei*, infections with various pathogens including WSSV have caused large economic losses. Viral pathogens are estimated to account for approximately 60% of disease losses, whereas bacterial pathogens account for approximately 20% (15). WSSV is the pathogen that most severely affects the sustainable development of the shrimp aquaculture industry, causing a high and rapid fatality rate (15). Therefore, exploring the pathogenic mechanism of WSSV in *L. vannamei* is particularly important. The hepatopancreas is a crucial organ in the immune system of *L. vannamei* (16). Therefore, we conducted transcriptomic and metabolomic analysis of the hepatopancreas of *L. vannamei* at different times after WSSV challenge.

### 4.1 Hepatopancreatic Transcriptomic Response After WSSV Challenge

On the basis of the published genome sequence of *L. vannamei*, transcriptomic analysis of the hepatopancreas of *L. vannamei* at different times after the WSSV challenge is important (17). We performed dynamic transcriptomic analysis of the hepatopancreas to better understand the molecular toxicity mechanism after WSSV challenge in *L. vannamei*. In this study, both up-regulated genes and down-regulated genes were identified in P2, P36 and P48. Similarly, in the DEGs of the three groups, many more down-regulated genes were found than up-regulated genes. The results implied that the gene expression changes in the hepatopancreas of *L. vannamei* were largely affected by WSSV challenge, which may impaired the immune function of the shrimp and cause immune dysregulation of the body of the shrimp.

Unlike vertebrates, the innate immune system of crustaceans is the main mean to combat microorganisms and pathogens (18). Tetraspanin is highly involved in viral and bacterial infections, plays a key role in pathogen adhesion to host cells and is important in the innate immune response in Macrobrachium rosenbergii (19). Our data showed that after WSSV challenge, the expression of tetraspanin-3-like in the hepatopancreas of *L. vannamei* was significantly down-regulated, in agreement with previous research (20). Chitinase helps crustaceans digest chitin in food and ensures the smooth shelling of crustaceans (21). Previous studies have shown that chitinase plays an important role in the innate immune response (21). In this study, chitinase-related genes (chitinase-3-like protein 1 and acidic mammalian chitinase-like) were significantly down-regulated in the three experimental groups as a result of immunosuppression. After invasion of pathogenic microorganisms, the first and most important step of the innate immune response in crustaceans is the identification of pathogen associated molecular patterns on the pathogen surface through various pattern recognition receptors (22), such as C-type lectin, which plays an important role in the innate immune response in crustaceans (23). Several lectin-related DEGs were identified as down-regulated genes in this study, including C-type lectin domain family 6 member A-like and ladder-like lectin in the three experimental groups. C-type lectin domain family 4 member G-like, transcript variant X1 in N-P36 and C-type lectin domain family 7 member A-like were identified in both N-P2 and N-P36. Notably, perlucin-like protein, a typical C-type lectin, was also down-regulated in N-P2 and N-P36. The CP450 cytochrome enzyme system, one of the main metabolic systems in the hepatopancreas, is widely involved in the metabolic processing of environmental toxins (such as WSSV), exogenous drugs, endogenous hormones and carcinogens (24). G-protein coupled receptors (GPCRs) respond to various stimuli and activate a series of signaling pathways in cells (25). Mucin regulates immunity, protecting the intestine and preventing damage by pathogenic bacteria (26). Cytochrome P450-related genes (cytochrome P450 2L1-like, transcript variant X2 and cytochrome P450 4C1-like), GPCR-related genes (G-protein coupled receptor Mth-like) and mucin-related genes (mucin-5AC-like) were down-regulated in the three experimental groups. The expression of some immune-related genes, such as crustacyanin-A1 subunit-like, esterase FE4-like, carbohydrate sulfotransferase 3-like, rap1 GTPase-activating protein 1-like, integrin alpha-4-like, cathepsin L1-like, trypsin-1-like, legumain-like, low-density lipoprotein receptor-like and RING finger protein 37-like, significantly changed.

Moreover, KEGG pathway enrichment analysis showed that most of the DEGs were significantly enriched in immune response pathways, such as pancreatic secretion, neuroactive ligand-receptor interaction, lysosome and protein digestion and absorption. Phospholipase A2 is a potential biomarker of inflammation that plays an important role in inflammatory diseases (27). The metal-binding protein carboxypeptidase participates in the immune response in *Mice* by activating or degrading signal molecules (28). Chen et al. found that the laccase in *L. vannamei* participates not only in immune defense but also in oxidative stress (29). Calcium-activated chloride channel regulator 1-like and calcium-independent phospholipase A2-gamma-like were differentially expressed in only N-P2, carboxypeptidase B-like was differentially expressed in N-P36, laccase-1-like was differentially expressed in N-P48, thus suggesting that the expression of these DEGs was time dependent. In crustaceans, peritrophin is considered a protective barrier for midgut epithelial cells. Elvin et al. found that in *Lucilia cuprina*, peritrophin plays an important role in preventing invasion by bacteria, viruses and other parasites (30, 31). Peritrophin-1-like was differentially expressed in only the P2, compared with the N group, thus indicating that the expression of peritrophin in the hepatopancreas of *L. vannamei* significantly differed in early stages after WSSV challenge. In crustaceans, hemocyanin is a respiratory protein in the hemolymph. Cheng et al. found that in *M. rosenbergii*, hemocyanin is mainly responsible for oxygen binding and carbon dioxide transport (32, 33). Hemocyanin C chain-like was down-regulated in only P2 and P48, compared with the N group, and the content of hemocyanin was reduced, thus indicating that WSSV challenge may cause hypoxia in the organism. In addition, other immune-related DEGs, such as pancreatic lipase-related protein 2-like, beta-1,3-glucan-binding protein, serine protease inhibitor dipetalogastin-like, WD repeat-containing protein 47-like, transcript variant X1 and leukocyte elastase inhibitor-like were down-regulated. Antibacterial peptides are small peptides with antibacterial activity (34). In this study, antibacterial peptides including lysozyme-like, astacin-like, anti-lipopolysaccharide factor-like and chitinase-3-like protein 1 were down-regulated. The invasion of WSSV destroyed the immune system of *L. vannamei*, thereby leading to the down-regulation of immune-related genes.

Apoptosis is believed to play an important role in normal life processes, and it can be triggered by a variety of molecular signals, thereby activating external and/or internal programmed cell death pathways (35). In many cases, host apoptosis inhibits viral replication, and simultaneously, viruses often express proteins that block the host’s apoptotic response (35). Caspases are an important family in the immune response in animals and are considered an indispensable part of the body’s response to environmental stimuli (36). In this study, apoptosis-related genes (caspase-1-A-like, cholinesterase 1-like, programmed cell death protein 6-like and zonadhesin-like) were significantly down-regulated in the three experimental groups. This finding indicated that the invasion of WSSV damaged the apoptotic system in shrimp, thus facilitating further expansion of WSSV.

The differential expression of cytoskeleton-related genes is also an important means through which shrimp cope with invasion by foreign pathogenic microorganisms (37). The shell and gastrointestinal tract in shrimp are covered with a cuticle, which provides the first line of defense against physical or chemical damage, and pathogen infection (38). Therefore, if WSSV virus particles enter the shrimp’s body, the cuticle first bears the brunt of the infection (38). In this study, cytoskeleton-related DEGs including actin-2, muscle-specific-like and transcript variant X2 were significantly up-regulated in the three experimental groups. The DEGs that were up-regulated included actin-like, actin, muscle-like, cuticle protein 7-like, cuticle protein CP1158-like, myosin regulatory light chain 2-like, transmembrane protein 53-like and tubulin–tyrosine ligase-like protein 12, transcript variant X1 in N-P2; actin-3, muscle-specific-like, keratin, type I cytoskeletal 10-like, keratin-associated protein 19-2-like, myoferlin-like, myosin heavy chain, muscle-like, pupal cuticle protein 36a-like and transmembrane protein 53-B-like in N-P36; and actin-2, muscle-specific-like, cuticle protein 7-like, transcript variant X1, cuticle protein AMP1A-like, cuticle protein CP14.6-like, cuticular protein 47Eg-like, keratin-associated protein 16–1-like, protein Skeletor, isoforms B/C-like, pupal cuticle protein Edg-84A-like and troponin C-like, transcript variant X1 in N-P48. The results indicated that the up-regulation of cytoskeleton remodeling-associated genes expression may confer stress protection and allow shrimp to respond to pathogen invasion or drastic environmental changes, in agreement with findings from previous studies (37). Moreover, we found that the expression of cytoskeleton-related genes showed temporal differences. The key genes for cytoskeleton remodeling in *L. vannamei* are different at different time points after being challenged by WSSV. In the P2 group, the expression of myosin regulatory light chain 2-like was significantly up-regulated, indicating that myosin regulatory light chain 2-like played a vital role in the initial stage of shrimp infection with WSSV. In the P36 group, the expression of actin-3, muscle-specific-like was significantly up-regulated, indicating that actin-3, muscle-specific-like was a key gene for cytoskeleton remodeling in shrimp 36 hours after infection with WSSV. In the P48 group, the expression of keratin-associated protein 16-1-like was significantly up-regulated, indicating that keratin-associated protein 16-1-like played an important role in the late stage of shrimp infection with WSSV. However, how these DEGs participate in the remodeling of the cytoskeleton requires further research.

In crustaceans, particularly aquatic animals, the production of reactive oxygen species is part of the body’s normal aerobic metabolism, which plays an important role in the immune system (1). However, excessive reactive oxygen species cause the body to produce oxidative stress, thus leading to cell oxidative damage (37). Therefore, to address the cell damage caused by oxidative stress, a powerful antioxidant system is required. This complex antioxidant protection system is mainly composed of peroxisomal, superoxide dismutase (SOD), catalase (CAT), thioredoxin (TRX), glutathione S-transferase (GST), GTP cyclohydrolase, metallothionein and phenoloxidase (37). In this study, peroxisomal N(1)-acetyl-spermine/spermidine oxidase-like and superoxide dismutase [Cu-Zn]-like, transcript variant X3 were significantly down-regulated in the three experimental groups. Notably, astacin-like, peroxisomal sarcosine oxidase-like, glutathione S-transferase 1-like and metallothionein-1-like were significantly down-regulated in N-P2; peroxisomal N(1)-acetyl-spermine/spermidine oxidase-like was significantly down-regulated in N-P36; and glutathione S-transferase 1-like, transcript variant X2 and glutathione S-transferase Mu 1-like were down-regulated in N-P48. These findings were consistent with the results of Parrilla (2013) indicating that the activity of various antioxidant enzymes in shrimps significantly decreases 48 h after WSSV infection (39). Simultaneously, the expression of these DEGs differed over time, and a series of DEGs were found to play an important and uninterrupted role in different time periods after WSSV challenge.

In addition, phagocytosis-related genes (vascular endothelial growth factor receptor 2-like, transcript variant X1) (40), detoxification-related genes (D-beta-hydroxybutyrate dehydrogenase, mitochondrial-like and liver carboxylesterase 2-like) (41), coagulation-related genes (coagulation factor IX-like, proclotting enzyme-like and multiple coagulation factor deficiency protein 2 homolog) (42), energy metabolism-associated genes (6-phosphofructo-2-kinase/fructose-2,6-bisphosphatase-like, transcript variant X1, phospholipase D alpha 1-like, transcript variant X3, UDP-glucose 4-epimerase-like, glyceraldehyde-3-phosphate dehydrogenase-like and succinate dehydrogenase [ubiquinone] iron-sulfur subunit, mitochondrial-like), the heat shock protein family (heat shock 70 kDa protein cognate 4-like, heat shock protein 60A-like, heat shock protein 83-like, heat shock protein HSP 90-alpha, transcript variant X1, hsp70-binding protein 1-like and heat shock 70 kDa protein cognate 4-like) (43) and the prophenoloxidase system (phenoloxidase-activating factor 3-like, phenoloxidase-activating factor 2-like and phenoloxidase-activating factor 1-like) (34, 44) were differentially expressed. These genes play crucial roles in regulating the shrimp response to physical and chemical factors, as well as pathogenic microorganisms in the environment. Furthermore, vascular endothelial growth factor receptor 2-like, transcript variant X1 (VEGF) was significantly up-regulated in N-P2. VEGF, a key component of the VEGF pathway, plays an important role in the formation of endothelial cells. Its up-regulation indicated that VEGF plays a crucial role in addressing pre-infection with WSSV, in agreement with results from previous studies (40).

### 4.2 Hepatopancreatic Metabolomic Response After WSSV Challenge

In this study, non-targeted metabolomics was used to analyze the DMs in the hepatopancreas of *L. vannamei* at different times after WSSV challenge. Compared with the N group, the three experimental groups (P2, P36 and P48) had 353, 332 and 405 DMs, respectively. The metabolomic data showed that WSSV challenge caused a series of metabolic disorders in the hepatopancreas of *L. vannamei*, including amino acid metabolism, lipid metabolism and nucleotide metabolism.

The metabolomic data also showed that some amino acids in the hepatopancreas of *L. vannamei* changed significantly after WSSV challenge. Among them, citrulline, glycitein, lysinoalanine, L-Serine and serylserine were down-regulated in the three experimental groups; valyl-tryptophan and L-histidine were down-regulated in P2; D-ornithine, L-leucyl-L-alanine and cysteinyl-proline were down-regulated in P36; and N-docosahexaenoyl histidine, arginyl-methionine, N-acetylasparagine and aspartyl-gamma-glutamate were down-regulated in P48. Alanine, tyrosine, serine, lysine and proline are all important intermediates of the tricarboxylic acid cycle, and important precursors of acetyl-CoA, oxaloacetate and α-ketoglutarate (45, 46). Ren’s study found that the corresponding amino acids in the hepatopancreas of *Marsupenaeus japonicus* under cold stress were differentially changed (46). Metabolites such as valine and leucine are involved in a series of biological functions such as immune defense, neurotransmission, energy production and protein synthesis (47). In this study, the significantly lower histidine, ornithine and asparagine in the three experimental groups than in the N group indicated that these amino acids might be used for energy metabolism. Marine mollusks usually use amino acids as an important energy source (48). Therefore, the decrease in amino acids in the shrimp hepatopancreas after WSSV challenge may indicate that amino acids were used as energy sources for the immune response of the shrimp against WSSV invasion (49). The significant changes in these amino acids perturbed the amino acid metabolism in the shrimp hepatopancreas, and were clearly caused by the invasion of WSSV.

In lipid metabolism, lipids mainly provide energy storage and supply in organisms, and simultaneously have a variety of biological functions in cell structure and biological processes (45). Arachidonic acid was up-regulated in P2 compared with the N group. Arachidonic acid is an essential fatty acid involved in energy supply, immune stress and nutritional supplementation (45). In addition, certain fatty acid levels associated with the regulation of immune cell function, such as 1-arachidonoylglycerophosphoinositol and docosatrienoic acid (P2, P36 and P48), were up-regulated, in agreement with previous studies (46, 50). Glycerophospholipids [phosphatidylcholine (PC), phosphatidylethanolamine (PE) and phosphatidic acid (PA)] are important parts of cell membranes and are the basis of signal transmission, cell metabolism and energy metabolism (46). In this study, lysophospholipids (LysoPC, LysoPE and LysoPA), raw materials for biofilms, were up-regulated in all three experimental groups. Among them, LysoPC influences the immune system by regulating immune cells (46). Therefore, we inferred that after WSSV infection, two reasons explain the general up-regulation of lipid metabolism in the hepatopancreas of *L. vannamei*: one is to provide an energy source for the shrimp to respond to WSSV invasion, and the other is to increase the level of immune-related metabolites to enhance the immunity of shrimp.

Nucleotide metabolism is one of the most important metabolic mechanisms in organisms (45). The data indicated that multiple nucleotide-related DMs were down-regulated in the three experimental groups. Among them, adenosine-related metabolites (guanosine diphosphate adenosine, diadenosine diphosphate, flavin adenine dinucleotide (FAD), adenine and N6-(delta2-isopentenyl)-adenine) mainly participate in cell respiration and energy synthesis, in agreement with previous findings (50). Guanosine and taurine inhibit cell apoptosis (51). In this study, the levels of 3-deoxyguanosine, deoxyguanosine, 7-methylguanosine 5’-diphosphate and N-stearoyl taurine were up-regulated after WSSV challenge, thus indicating that WSSV invasion may cause spontaneous anti-apoptosis in prawns. In conclusion, we inferred that the invasion of WSSV perturbed nucleotide metabolism in the hepatopancreas of *L. vannamei*.

### 4.3 Combined Response of Hepatopancreas Transcriptomics and Metabolomics After WSSV Challenge

There was a correlation between the DEGs and DMs of the shrimp hepatopancreas after 2h, 36h and 48h of WSSV challenge in *L. vannamei*. In the P2 group, compared with the N group, As a down-regulated DEG, cystathionine beta-synthase-like, transcript variant X1 plays a vital role in the cysteine and methionine metabolism pathway. The down-regulation of cystathionine beta-synthase-like, transcript variant X1 leads to the down-regulation of the expression level of the L-Serine, indicating that there is a positive correlation between them. Group XV phospholipase A2-like is a metabolism related genes in the glycerophospholipid metabolism pathway, and its down-regulation leads to the up-regulated expression of LysoPC(22:6(4Z,7Z,10Z,13Z,16Z,19Z)), which indicates that there is a negative correlation between them. In the P36 group, compared with the N group, in the neuroactive ligand-receptor interaction metabolic pathway, the up-regulation of the expression level of trypsin-1-like leads to the up-regulation of acetylcholine and anandamide in this metabolic pathway, which indicates that there is a positive correlation between them. In the P48 group, compared with the N group, fatty aldehyde dehydrogenase-like is a metabolism related genes in the tyrosine metabolism, and its down-regulation leads to the down-regulated expression of dopamine, which indicates that there is a positive correlation between them. There are complex and huge metabolic pathways in organisms, and changes in the expression levels of certain metabolites may be regulated by changes in single or multiple genes. Therefore, the relationship between DEGs and DMs needs to be further studied.

## 5 Conclusion

In conclusion, we studied the changes in the transcriptome and metabolome in the hepatopancreas of *L. vannamei* after 2 h, 36 h and 48 h of WSSV challenge. In terms of the transcriptomic changes, immune, apoptosis, cytoskeleton and antioxidant-related genes were observed. Genes associated with phagocytosis, detoxification, blood coagulation and energy metabolism were also found to be involved. The metabolomic analysis mainly revealed differential amino acid metabolism, lipid metabolism and nucleotide metabolism. In summary, the invasion by WSSV led to differences in gene expression levels in shrimp, which in turn led to differences in the levels of metabolites. These DEGs and DMs provide a reference for understanding the pathogenic mechanism of WSSV at the molecular level.

## Data Availability Statement

The datasets presented in this study can be found in online repositories. The names of the repository/repositories and accession number(s) can be found below: BioProject is PRJNA773857, SRR16544406, SRR16544405, SRR16544404, SRR16544403, SRR16544402, SRR16544401, SRR16544400, SRR16544399, SRR16544398, SRR16544397, SRR16544396, and SRR16544395.

## Author Contributions

DY designed and performed the experiments, data analysis, and wrote the manuscript. YZ designed and performed the experiments and data analysis. PH assisted in the experimental design. RJ assisted in the experimental design, and edited of the manuscript. DY and YZ contributed to the sampling and data analysis. All authors contributed to the article and approved the submitted version.

## Funding

This research was supported by the National Key Research and Development Plan (2019YFC0312604), the Shanghai Agriculture Science and Technology Innovation Project (2017, No 1–13), the Shanghai Science and Technology Commission Innovation Project (Nos. 16391903500, and 17391902200), and the National Marine 863 Project (No. 2014AA093506).

## Conflict of Interest

The authors declare that the research was conducted in the absence of any commercial or financial relationships that could be construed as a potential conflict of interest.

## Publisher’s Note

All claims expressed in this article are solely those of the authors and do not necessarily represent those of their affiliated organizations, or those of the publisher, the editors and the reviewers. Any product that may be evaluated in this article, or claim that may be made by its manufacturer, is not guaranteed or endorsed by the publisher.
