# Supplementary figures and images for: Comprehensive Transcriptomic and Metabolomic Analysis of the Litopenaeus vannamei Hepatopancreas After WSSV Challenge
Source: Front Immunol. 2022 Feb 10;13:826794. doi: 10.3389/fimmu.2022.826794 (PMC8867067; doi:10.3389/fimmu.2022.826794)

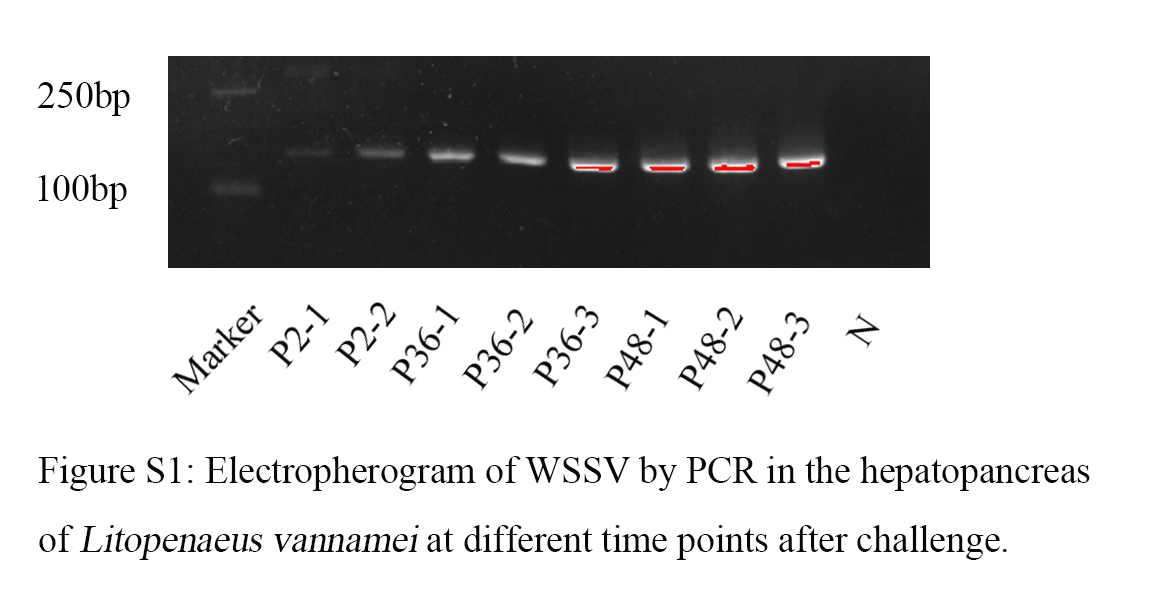

Supplement: Supplementary file 1 [file Image_1.tif]

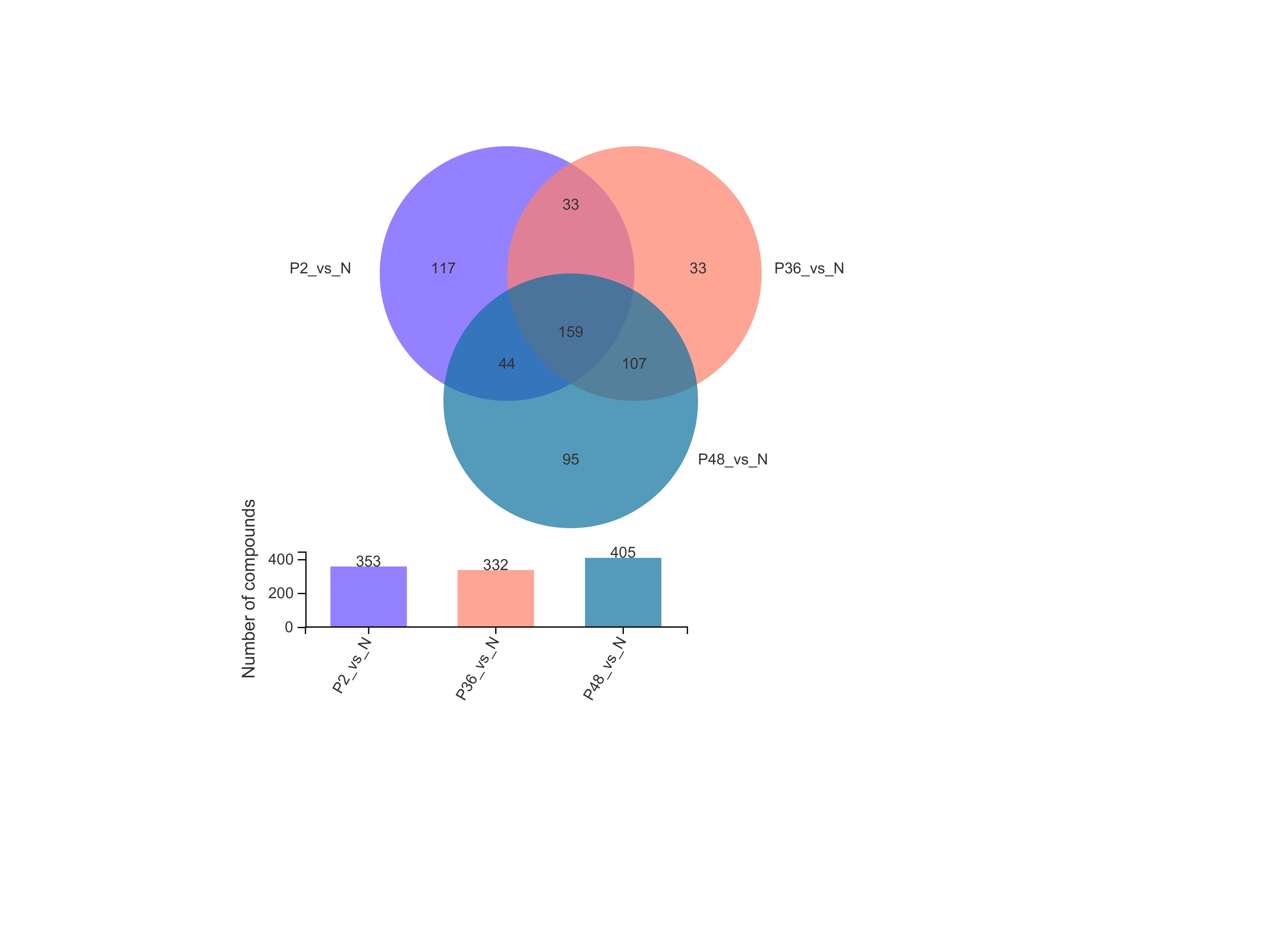

Supplement: Supplementary file 2 [file Image_2.tif]
